# Simulation-Based Estimates of the Effectiveness and Cost-Effectiveness of Pulmonary Rehabilitation in Patients with Chronic Obstructive Pulmonary Disease in France

**DOI:** 10.1371/journal.pone.0156514

**Published:** 2016-06-21

**Authors:** Kokuvi Atsou, Perrine Crequit, Christos Chouaid, Gilles Hejblum

**Affiliations:** 1 INSERM, UMR_S 1136, Institut Pierre Louis d'Epidémiologie et de Santé Publique, Paris, Sorbonne Universités; 2 UPMC Univ Paris 06, AP-HP, Hôpital Saint-Antoine, Unité de Santé Publique, Paris, France; 3 GRC OncoTho, Paris Est, UPEC, Créteil, CHI Créteil, Service de Pneumologie, Créteil, France; University of Athens, GREECE

## Abstract

**Background:**

The medico-economic impact of pulmonary rehabilitation in patients with chronic obstructive pulmonary disease (COPD) is poorly documented.

**Objective:**

To estimate the effectiveness and cost-effectiveness of pulmonary rehabilitation in a hypothetical cohort of COPD patients.

**Methods:**

We used a multi-state Markov model, adopting society’s perspective. Simulated cohorts of French GOLD stage 2 to 4 COPD patients with and without pulmonary rehabilitation were compared in terms of life expectancy, quality-adjusted life years (QALY), disease-related costs, and the incremental cost-effectiveness ratio (ICER). Sensitivity analyses included variations of key model parameters.

**Principal Findings:**

At the horizon of a COPD patient’s remaining lifetime, pulmonary rehabilitation would result in mean gain of 0.8 QALY, with an over disease-related costs of 14 102 € per patient. The ICER was 17 583 €/QALY. Sensitivity analysis showed that pulmonary rehabilitation was cost-effective in every scenario (ICER <50 000 €/QALY).

**Conclusions:**

These results should provide a useful basis for COPD pulmonary rehabilitation programs.

## Introduction

Chronic obstructive pulmonary disease (COPD) is a common and costly disease worldwide, and the associated disease burden is projected to rise [1]. Pulmonary rehabilitation (PR) is an essential part of the management of individuals with COPD [2]. PR consists of a multidisciplinary program that includes patient assessment, exercise training, education, nutritional advice, smoking cessation interventions, and psychosocial support. Exercise training is the component for which evidence of benefit is strongest. Therefore, when resources are scarce, the priority is to offer a program of supervised exercise training. PR may be conducted at home, or on an outpatient or inpatient basis. The evidence supporting PR for COPD patients is compelling [2–7]. Benefits include a decrease in symptoms (dyspnoea, fatigue, anxiety and depression), improved exercise tolerance, and better health-related quality of life (HRQoL) [8–10]. These benefits have been shown both in patients with stable disease and in those recovering from an acute exacerbation [2, 11–13]. PR is thus recommended in international guidelines for the management of patients with moderate to severe COPD [2].

In contrast, the economic impact of PR is poorly documented. Estimates of the cost of running a PR program have rarely been reported [14–15]. These costs depend on the local health care system, existing infrastructure and equipment, staff requirements, and the duration of the program. Low-cost programs in existing facilities have been shown to be effective [16–17]. Likewise, PR significantly reduces the direct costs of COPD by avoiding unnecessary use of the healthcare system, particularly unplanned hospital admissions. This also appears to hold true when PR is initiated immediately after a COPD exacerbation. However, contrary to other COPD interventions, whether therapeutic (bronchodilators, corticosteroids) or non-therapeutic, the cost-effectiveness of PR for COPD has rarely been evaluated in prospective randomized studies [18–21]. In addition, available studies used a limited time horizon (usually one year) and could not therefore determine long-term cost-effectiveness.

The aim of this study, based on published data, was to obtain simulation-based estimates of the effectiveness and cost-effectiveness of PR in a hypothetical cohort of COPD patients.

## Patients and Methods

The effectiveness and cost-effectiveness of PR in patients with COPD were estimated by using a Markov model (Tree-Age Pro software Inc. Williamstown, MA, USA, 2009) to describe the outcomes of a French cohort of COPD patients with and without PR (one course every 2 years, for lifespan). Society’s viewpoint was adopted, with a time horizon corresponding to the patients' remaining lifespan. The results of the simulation are expressed in life years (LY), quality-adjusted life years (QALY) and costs of care (2015 euros, with a discount of 3.5% per year). The incremental cost-effectiveness ratio (ICER) of PR was calculated as the ratio of the difference in effectiveness and in costs for COPD patients managed with and without PR.

### Modelling

**Fig 1:**
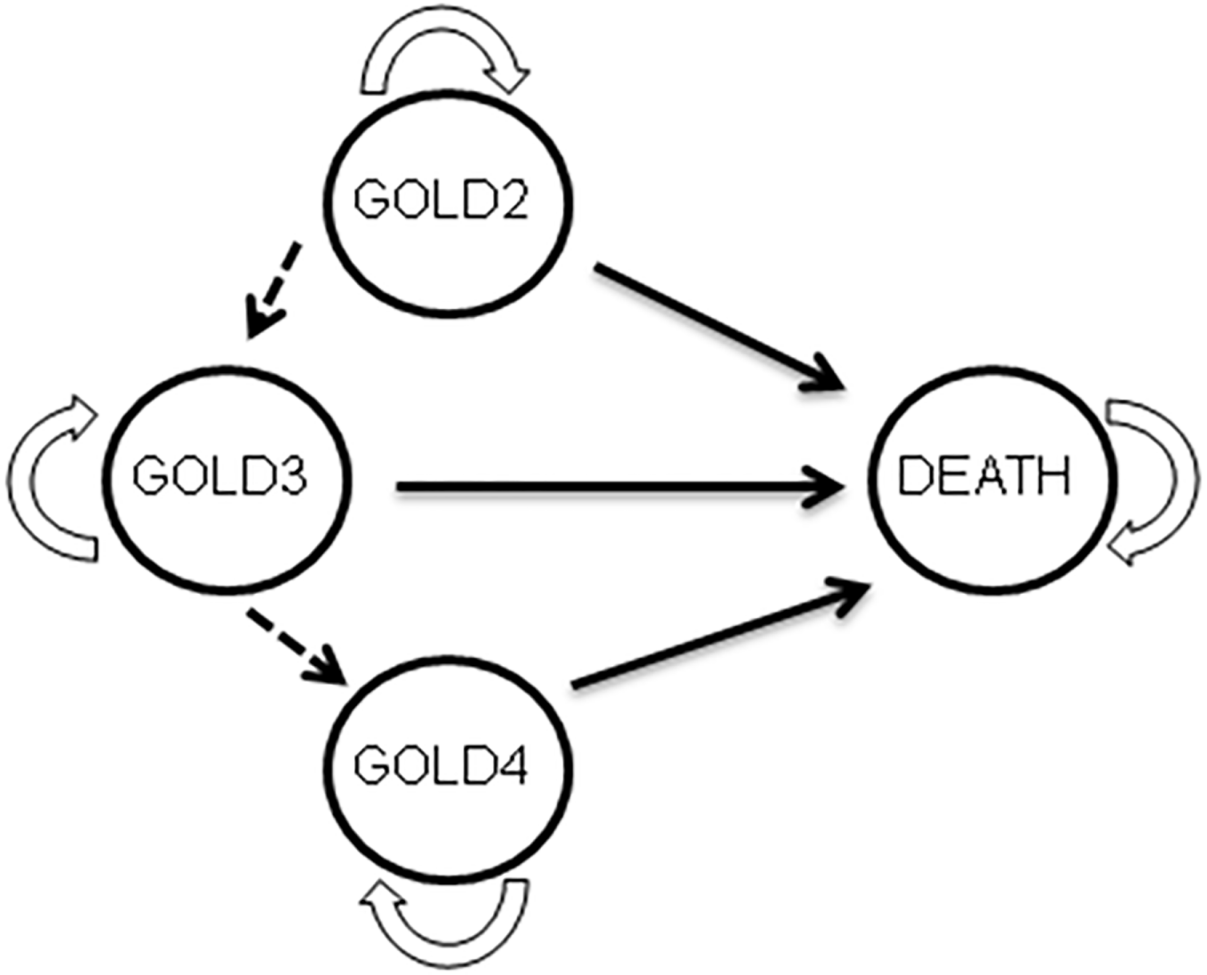
Decision model. Four health states (GOLD2 to GOLD4 and death) defining the outcome of a patient with COPD. The arrows indicate the possibility of transition from one state to another. Transition from one state to another, based on GOLD criteria, is unidirectional. GOLD4 patients cannot transit to another stage, and « death » is an absorbing state in which transition to another state is not possible.

The model used here is described in detail elsewhere [22]. Briefly (Fig 1), each patient was initially placed in one of the following three health states defining COPD severity: GOLD2, GOLD3 or GOLD4 (GOLD1 patients were not considered here, as PR is not indicated at this stage). Each patient was assigned an age, smoking status (smoker or non-smoker) and a risk of exacerbation [22]. The time horizon of the simulation was divided into one-year time steps (cycles). At each cycle the smoking status of each patient could be modified (cessation among smokers or resumption among ex-smokers). Similarly, depending on their number of previous exacerbations and health state, patients could have one or more exacerbations. At each cycle, each patient remained at the same stage, progressed to a more serious stage, or died; the transition probabilities between health states depended on the current health state, age, smoking status, and whether or not exacerbations occurred. Specific mortality tables were built using three information sources: all-cause mortality data for the UK general population in 2007, cohort data taking into account excess mortality associated with COPD, and cohort data taking into account the smoking status of COPD patients [22].

### Specific Parameters Related to PR

In order to implement the model, we conducted a structured literature search focusing on original articles, reviews and meta-analyses published in French or English between January 2009 and 28 June 2014 and reporting data on improvements in exacerbation rates and quality of life (SGRQ total score) of COPD patients participating in PR programs (S1 Fig). This analysis (S1 Table) showed that PR improved quality of life in the vast majority of prospective randomized trials (average improvement of 7.6 units on the SGRQ total score, or 0.09 QALYs according to Oba et al. [23]. Therefore, in the model, we allowed an additional utility of 0.09 for PR, whatever the severity stage. The impact of PR on mortality among COPD patients is less well established. The majority of studies showed no impact, one study showed a non-significant trend towards a reduction, and three others showed statistically significant reductions (S1 Table). Similarly, apart from one study that showed a 46% decrease, no effect of PR on the rate of exacerbations was found (S1 Table). We adopted a conservative perspective: in the baseline scenario, PR was considered not to affect the rate of exacerbations, mortality, or the smoking cessation rate (S2 Table).

### Parameter Values of the Model

Table 1 reported the demographic characteristics, the prevalence of each severity stage, the odds of smoking cessation, the likelihood of exacerbations in a given patient at a given severity stage, and the management costs of COPD. These parameters were based on French observational cohorts of COPD patients [24–26]. The costs of COPD, available for each severity stage, were limited to direct medical costs (medications, physician visits and consultations, laboratory tests and investigations, respiratory support, nursing sessions, physical therapy, hospitalization), non-medical costs (medical transport), and work stoppages. We excluded indirect costs (e.g. lost productivity). Costs of a PR course included all direct costs (physiotherapy, medical, nurse and administrative costs).

**Table 1:** characteristics of the population without pulmonary rehabilitation (usual cares) and with pulmonary rehabilitation (PR); ^+^% non smokers/ex-smokers/smokers; ^++^% of patients at least 1 exacerbation per year.

	Usual Cares	PR	Ref
**Distribution according to severity (%patients)**			[24]
GOLD2	88.3	88.3	
GOLD3	10.5	10.5	
GOLD4	1.2	1.2	
**Smoking initial status+**			[24]
GOLD2	12.5/62.2 /25.3	12.5/62.2 /25.3	
GOLD3	10.2/63.6/26.3	10.2/63.6/26.3	
GOLD4	8.7/71.4/19.9	8.7/71.4/19.9	
**Smoking status turnover (% patients each)**			[30]
smoking cessation (% smokers)	4.7	4.7	
smoking relapse (% ex-smokers)	2.6	2.6	
**Age distribution according to severity**	Table S1	Table S1	[22]
**Transition probability from one stage to another**	Table S2	Table S2	[22]
**Probability of death according to COPD stage**	Table S3	Table S3	[22]
**Rate of exacerbations**^**++**^			[28]
GOLD2	39.45	39.45	
GOLD3	44.1	44.1	
GOLD4	66.7	66.7	
**Heath utilities (0 / at least 1 exacerbation)**			[27]
GOLD2	0.755; 0.736	0.755; 0.736	
GOLD3	0.748; 0.726	0.748; 0.726	
GOLD4	0.549; 0.535	0.549; 0.535	
**Additional utility due to rehabilitation**		0.09	
**Heath costs (€ per patient and per year)**			[25]
GOLD2	5398	5398	
GOLD3	5567	5567	
GOLD4	10953	10953	
**Additional cost for PR (€ per patient per year)**		1583	[26]

The cost for one PR course was 1583 euros (administrative costs, medical fees, physiotherapy costs and non-medical fees representing respectively 37.1%, 10.7%, 29.1% and 23.1% of this cost), corresponding to the average cost of these programs in France, where, depending on health status, a patient is managed as an outpatient, at home, in a day hospital or, occasionally, in hospital [26].

Utilities were derived from the work of Szende et al. [27] and were 0.7511, 0.7481 and 0.5493 respectively for stable (no exacerbations for one year) GOLD stage 2, 3 and 4 patients and 0.7364 0.7261 and 0.5357 respectively for GOLD stage 2, 3 and 4 patients with at least one exacerbation. The rate of exacerbations [28] depended on the severity stage. Progression from one severity stage to another over time was based on data from the Framingham cohort [29]. Transition probabilities from one stage to another depended on smoking status [30].

### Assessing Uncertainty

The uncertainty in the model was evaluated by means of one-way sensitivity analysis. The estimate for a given model parameter was varied, while keeping the other parameters constant, within a range of likely values derived from confidence intervals or reasonable ranges collected from published sources. Scenario-based sensitivity analyses explored the impact of changes in the model parameter values, including a change in the value of health states (variation between the limits of the 95% confidence interval of the mean), a discount of 5% and a discount of 50% every two years for the gain allowed by a course of PR, as well as the cost of care of COPD patients (5% and 10%), the cost of PR (±50%), and a reduction in the rate of exacerbations (up to 46%, the highest value found in the literature) [31]. Three simulations were used to examine the combined impact of these parameters. The first simulation considered that PR reduced the rate of exacerbations by 46% and COPD management costs by 10%; the second that PR led to a 46% decrease in the rate of exacerbations, associated with an improvement in health utilities of 0.125 (third quartile); and the third, that PR led to a 46% decrease in the rate of exacerbations, a 10% reduction in care costs, and an improvement in health utilities of 0.125.

Finally, we also conducted a multivariate probabilistic sensitivity analysis, implemented in a second-order Monte Carlo simulation. A simulation with 10 000 replications of the model was used to obtain the non-parametric 95% confidence intervals for the cost and effectiveness parameters. SAS software version 9 (SAS INC Cary NC) and the Data TreeAge Pro HealthCare program were used for statistical analyses and modelling, respectively.

## Results

**Fig 2:**
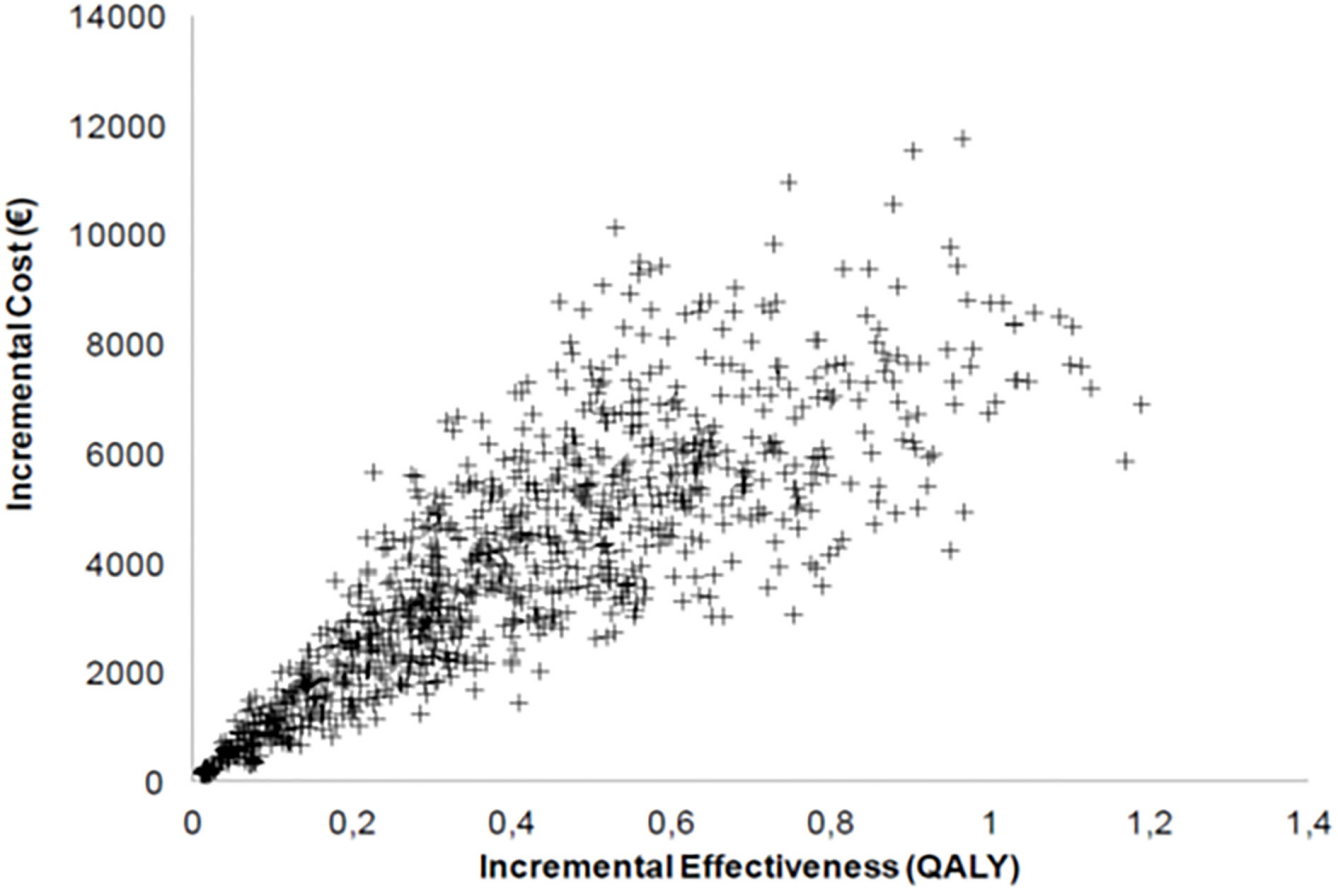
Probabilistic sensitivity analysis. Incremental costs (€) of pulmonary rehabilitation intervention as a function of its incremental effectiveness (QALY).

**Fig 3:**
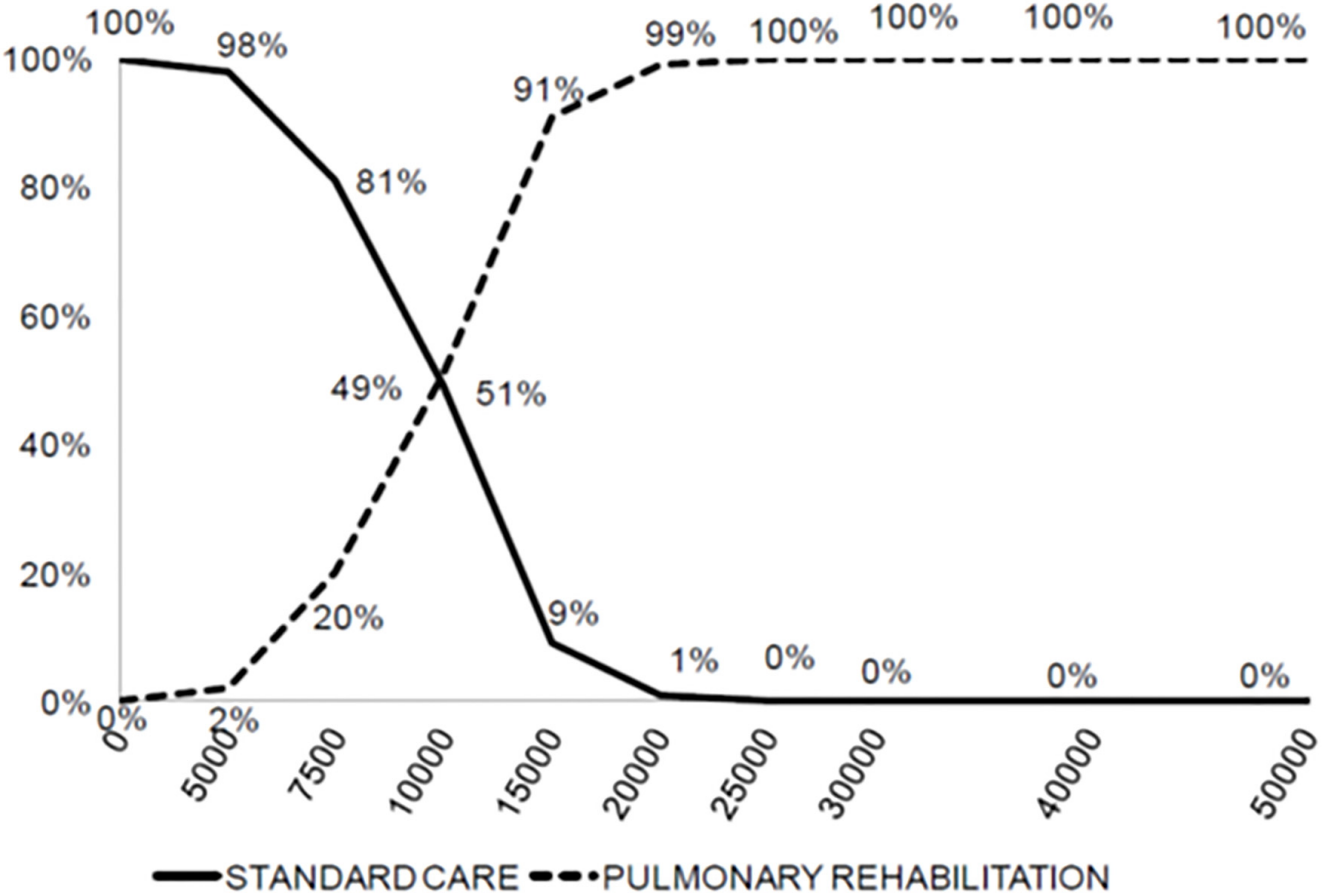
Acceptability curve. Percent chances that PR is-cost-effective (as compared to standard care) as a function of the willingness to pay (€/QALY) for it.

The results of the baseline scenario analysis are shown in Table 2. Without PR, the cost of care for the entire lifespan of a COPD patient was 72 993 euros for 16.6 LY and 8.4 QALY. PR yielded a gain of 0.8 QALYs at an incremental cost of € 14 102 per patient, with an ICER of € 17 583/QALY. The scenario-based sensitivity analyses (Table 3) showed that PR was cost-effective in most cases. The main driver was the additional utility allowed by PR with, for example an ICER of 52 750 euros per Qaly if the PR allowed a increase of 0.03 (compared to 0.09) in utility. The impact of a reduction in exacerbations allowed by PR is not major. If PR allowed a 46% reduction in exacerbations compared to the baseline case, there were more additional costs but also many more life years gained, and the ICER was better (15689 euros per Qaly, Table 3). A discount over time of the efficacy of PR increases the ICER. Fig 2 shows the results of multivariate probabilistic sensitivity analysis, with an ICER of <50 000 €/QALY in most cases. The acceptability curve (Fig 3) confirmed these results.

**Table 2:** Simulation result comparing Respiratory rehabilitation program to usual care (QALY: quality adjusted life year, ICER: incremental cost effectiveness ratio).

	Costs (€)	Life year	QALY	ICER
Usual care	72 993	16.608	8.395	
PR	87 095	16.608	8.485	
*Differences*	*14 102*	*0*	*0*.*802*	*17 583*

**Table 3:** Sensitivity analysis results (LY: life year, QALY: quality adjusted life year, ICER: incremental cost effectiveness ratio); Simulation 1: Exacerbation rate = -46% and Increase QALY = 0.125; Simulation 2: Exacerbation rate = -46% and COPD costs = -10%, Simulation 3: Exacerbation rate = -46% and COPD costs = -10% and Increase QALY = 0.125.

	Additional cost (€)	LY gained	QALY gained	ICER
*Baseline case*	14 102	0	0.8	17 583
*Increase QALY = 0*.*03*	14 102	0	0.267	52 750
*Increase QALY = 0*.*17*	14 102	0	1.515	9 309
Discount Qaly over time: 5%	14 102	0	0.77	18314
Discount Qaly over time: 50%	14102	0	0.72	17904
*Exacerbation rate = -46%*	18 533	0.962	1.181	15 689
*COPD costs = -5%*	10 453	0	0.802	13 033
*COPD costs = -10%*	6 803	0	0.802	8 482
*Rehabilitation Costs = -50%*	7 051	0	0.802	8 792
*Rehabilitation_Costs +50%*	21 154	0	0.802	26 375
Simulation 1	18 533	0.962	1.503	12 334
Simulation 2	10 834	0.962	1.181	9 171
Simulation 3	10 834	0.962	1.503	7 210

## Discussion

This simulation, conducted from society’s perspective and based on data for a COPD population managed in France throughout their lives, showed that pulmonary rehabilitation, with a ICER of € 17 583/QALY, was cost-effective, even in the conservative situation in which PR only enhanced quality of life, without improving the rate of smoking cessation, the frequency of exacerbations, or mortality. Published data on the cost of PR programs, their impact on healthcare consumption and their cost-effectiveness are very heterogeneous. Program costs depend on local organization (number of sessions, staffing, and the setting (ambulatory/outpatient/inpatient)). In a non-hospital environment, these costs range from £280.20 in the UK (14) to $1000 in the USA [15]. They are higher in the hospital environment [32]. The average cost retained in our baseline scenario (1583 euros) is entirely consistent with published data. Under the assumption that each patient has a course of PR every two years, this cost has a major impact on the ICER: when the cost changed by ±50%, the ICER changed from € 8792 to € 26 375 per QALY.

In non-randomized cohort studies, PR reduced healthcare consumption in both the short and long term [17, 18, 33, 24]. An Australian study [16] showed that an 8-week outpatient PR program reduced by 46% the number of patients admitted to hospital with a COPD exacerbation and by 62% the total number of bed-days in the 12 months following PR, by comparison with the previous year. Another economic evaluation of community-based PR showed a reduction in total annual costs of Can$ 344 per person [15], with significant reductions in exacerbations, hospitalization, and hospital bed-days, regardless of COPD severity (respectively -44%, -63%, and -55%; all p <.05). Griffiths et al [18] also showed a decrease in the number of home medical visits, hospitalizations, and hospital bed-days after a 6-week 18-course PR program. Other studies showed reductions in hospitalization rates and lengths of stay after self-management programs that included home exercises and some components of conventional rehabilitation [7].

The main limitation of these studies is the lack of randomized control groups and the existence of many confounding factors (changes in medication, improved management, etc.). Randomized studies are rarer [18–20, 31–35]. The most thorough study [18] randomly assigned 200 patients to a 6-week outpatient PR program or to ongoing standard medical management and prospectively collected costs and health utilities. Medical treatment was systematically optimized, and patients could be referred for smoking cessation counselling, dietary assessment, occupational therapy and physiotherapy if necessary. At the conclusion of the PR program, patients were invited to join a patient-run weekly group meeting at the local leisure centre. The program resulted in an increase of 0.03 QALYs per patient (p = 0.03) and a non-significant mean cost saving of £152 per patient. As in our study, the effectiveness acceptability curve showed that the ICER of the PR program was below £10 000 in 90% of cases, but as we did not have the same time horizon, it is difficult to compare our results with those of these studies.

An economic evaluation conducted alongside a randomised controlled trial of a low-intensity maintenance programme over a time horizon of 1 year delivered in UK primary and secondary care settings [36] showed that, at 12 months, the intervention was dominant (less costly and more effective) [21]. Another study from the Netherlands investigated the cost-effectiveness and cost utility of a community-based 20-month management scheme following 4 months of intensive PR [37]. The estimated ICER was €32 425 per QALY, and the estimated probability of cost effectiveness at a willingness-to-pay of €20 000 per QALY was 33%.

In an Irish setting [38], a cost-effectiveness analysis of an 8-week structured PR programme for COPD patients, with follow-up at 22 weeks, yielded an incremental cost of €472 000 per QALY gained, a value considerably above any ‘reasonable’ threshold. An RCT-based study of a 6-week programme of hospital- versus community-based PR, with and without telephone follow-up, in patients with COPD, showed a 50% probability of cost-effectiveness for hospital versus community PR at a £30 000-per-QALY threshold. Telephone follow-up appeared to improve outcomes at reasonable cost in the community-based group but not in the hospital group [34]. Our results are in line with the recently value placed on COPD interventions developed by the London Respiratory Network with the London School of Economics [39,40] In this analysis, PR intervention had an ICER between 2000 and 8000 £ per Qaly, very close of our model-based results. These results justify PR as add-on therapy to pharmacologic treatment for COPD patients.

Our study has certain limitations. For example, there is no agreed definition of exacerbations, and the proportion of patients with exacerbations in our literature analysis may have been incorrectly estimated. However, the sensitivity analyses indicated that PR remained cost-effective even when the proportion of exacerbation-free patients increased or when ex-smokers experienced fewer exacerbations. Other uncertainties concern the cost of PR programs. For the base case scenario, we used the average cost of French programs (ambulatory, inpatient or outpatient). This is clearly an important driver of the ICER for PR: low-cost ambulatory programs have a far better ICER than those relying on hospital structures. Also, parameter values required for the model had to be derived from studies performed in different countries, and the corresponding variability in COPD epidemiological parameters remains an unavoidable limitation [40]. Another limitation is that the relative frequencies of co-morbidities in COPD smokers and ex-smokers have not been reported, and neither have their associated specific costs [41]. As such costs could not be taken into account in the simulations, contrary to the corresponding health outcomes (the health utilities and all-cause death rates used in the simulations included co-morbidities), the cost-effectiveness estimates derived from the simulations should be considered as a lower limit: the increase in favour of the PR cost-effectiveness estimates when the costs of co-morbidities are taken into account remains to be determined.

Finally, we adopted a conservative perspective: in the baseline scenario, PR was considered not to affect the rate of exacerbations, mortality, or the smoking cessation rate, but we tested the impact of a change in these parameters by conducting a one-way sensitivity analysis.

## Conclusion

This study shows that PR is cost-effective, from society’s viewpoint, throughout COPD patients’ lifespan. Our model shows the lifespan benefit for patients and identifies the parameters that most affect related costs. These results represent a further argument for generalizing PR programs in which courses are repeated throughout the lifespan of COPD patients in order to maintain the gains thus achieved, in line with recent international guidelines [42].

## Supporting Information

S1 Fig Data search and selection flowchart. (DOCX)

S1 Table Impact of pulmonary rehabilitation on patients’ health. (DOCX)

S2 Table Parameter values use in the Marko simulation. (DOCX)
