# Supplementary material for: Simulation-Based Estimates of the Effectiveness and Cost-Effectiveness of Pulmonary Rehabilitation in Patients with Chronic Obstructive Pulmonary Disease in France
Source: PLoS One. 2016 Jun 21;11(6):e0156514. doi: 10.1371/journal.pone.0156514 (PMC4915708; doi:10.1371/journal.pone.0156514)
Supplement: S1 Fig — (DOCX) [file pone.0156514.s001.docx]

S1 Fig: Data search and selection flowchart

**
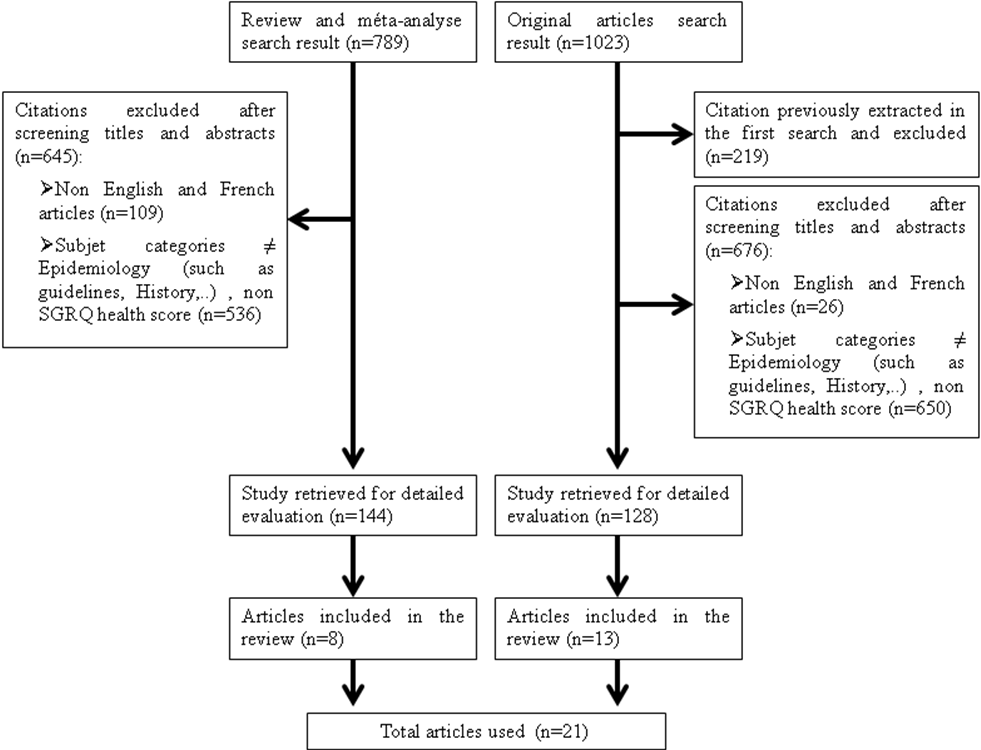
**

In order to implement the model with the impact of PR on the course of COPD, we made a structured literature search, using MEDLINE and focusing on original articles, reviews and meta-analyses published between January 2009 and 28 June 2014. The following MeSH search terms were used: "Pulmonary Disease, Chronic Obstructive"[Mesh]; "Pulmonary Disease, Chronic Obstructive/rehabilitation"[Mesh]; "Pulmonary Disease, Chronic Obstructive/nursing"[Mesh]; "Pulmonary Disease, Chronic Obstructive/prevention and control"[Mesh]; "Rehabilitation Nursing"[Mesh]; "Rehabilitation"[Mesh]; "rehabilitation" [Subheading]; fitness*; exercise*; physical*; train*.

We focused on papers published in French or English and reporting data on improvements in the frequency of exacerbations and quality of life (SGRQ total score) of COPD patients participating in pulmonary rehabilitation programs.
