# Supplementary material for: Simulation-Based Estimates of the Effectiveness and Cost-Effectiveness of Pulmonary Rehabilitation in Patients with Chronic Obstructive Pulmonary Disease in France
Source: PLoS One. 2016 Jun 21;11(6):e0156514. doi: 10.1371/journal.pone.0156514 (PMC4915708; doi:10.1371/journal.pone.0156514)
Supplement: S1 Table — (DOCX) [file pone.0156514.s002.docx]

S1 table 1: Impact of pulmonary rehabilitation on patient’s health

| References | Quality of life  (SGRQ total score) | Mortality | Exacerbations |
| --- | --- | --- | --- |
| Puhan M (1s) | -9.88 [-14.40. -5.37] | OR= 0.28 [0.10. 0.84] |  |
| Effing T (2s) | -2.58 [-5.14. -0.02] |  |  |
| Lacasse Y (3s) | -6.11 [-8.98. -3.24] |  |  |
| Vieira DS (4s) | -5.1 [-12.2; -4.4] |  |  |
| Peytremann-Bridevaux I (5s) |  | OR = 0.85 [0.54.1.36] | (3.7/6.9)* |
| Puhan MA (6s) | -11.1 [-17.1; -5.2] | OR = 0.45 [0.22;0.91] |  |
| Al-Ghimlas F (7s) | -6.71 [-13.89; -0.46] |  |  |
| [Zaina F](http://www.ncbi.nlm.nih.gov/pubmed?term=Zaina%20F%5BAuthor%5D&cauthor=true&cauthor_uid=19532107) (8s) | -9.9 [-18; -1.73] | OR = 0.29 [0.10;0.84] |  |
| Seymour JM (9s) | -8.2 (-15.1;-1.3). |  |  |
| Lan CC (10s) | -13.2 [-21.4; -5.1] |  |  |
| Dodd JW (11s) | -3.9(9.0)^†^ |  |  |
| Clini EM (12s) | -5.89 [-6.45; -5.34] |  |  |
| Bratås O (13s) | –3.1 [-5.1; -1.1] |  |  |
| Steinsbekk A (14s) | –9.4 (-1.4; -0.6) |  |  |
| Theander K (15s) | -7.6 (10.8) |  |  |
| Ninot G (16s) | –8 [-16; 0] |  |  |
| Spencer LM (17s) | -9. [-15; -4] |  |  |
| Beaumont M (18s) | -15.43 |  |  |
| Hoogendoorn M (19s) | 0.08 QALY [-0.01–0.18] |  |  |
| Esteban C (20s) | -5.3^#^ |  |  |

^*^Ratio between rehabilitated patients exacerbations and non rehabilitated exacerbations, over two years.

Estimates were reported in various ways: mean or mean (SD) or [95% confidence interval]

^#^ not reported

1s. Puhan M, Scharplatz M, Troosters T, Walters EH, Steurer J. Pulmonary rehabilitation following exacerbations of chronic obstructive pulmonary disease. Cochrane Database Syst Rev 2009;21:CD005305. Review. Update in: Cochrane Database Syst Rev 2011;10:CD005305.

2s. Effing T, Monninkhof EM, van der Valk PD, van der Palen J, van Herwaarden CL, Partidge MR, Walters EH, Zielhuis GA. [Self-management education for patients with chronic obstructive pulmonary disease.](http://www.ncbi.nlm.nih.gov/pubmed/17943778) Cochrane Database Syst Rev 2007;4:CD002990.

3s. Lacasse Y, Goldstein R, Lasserson TJ, Martin S. [Pulmonary rehabilitation for chronic obstructive pulmonary disease.](http://www.ncbi.nlm.nih.gov/pubmed/17054186) Cochrane Database Syst Rev 2006;:CD003793.

4s. Vieira DS, Maltais F, Bourbeau J. Home-based pulmonary rehabilitation in chronic obstructive pulmonary disease patients. Curr Opin Pulm Med 2010;16:134-43.

5s. Peytremann-Bridevaux I, Staeger P, Bridevaux PO, Ghali WA, Burnand B. Effectiveness of chronic obstructive pulmonary disease-management programs: systematic review and meta-analysis. Am J Med 2008;121:433-443.

6s. Puhan MA, Scharplatz M, Troosters T, Steurer J. [Respiratory rehabilitation after acute exacerbation of COPD may reduce risk for readmission and mortality -- a systematic review.](http://www.ncbi.nlm.nih.gov/pubmed/15943867) Respir Res 2005;6:54.

7s. Al-Ghimlas F, Todd DC. [Creatine supplementation for patients with COPD receiving pulmonary rehabilitation: a systematic review and meta-analysis.](http://www.ncbi.nlm.nih.gov/pubmed/20497386) Respirology 2010;15):785-95.

8s. [Zaina F](http://www.ncbi.nlm.nih.gov/pubmed?term=Zaina%20F%5BAuthor%5D&cauthor=true&cauthor_uid=19532107), [Negrini S](http://www.ncbi.nlm.nih.gov/pubmed?term=Negrini%20S%5BAuthor%5D&cauthor=true&cauthor_uid=19532107). EJPRM systematic continuous update on Cochrane reviews in rehabilitation: news from the first issue 2009. [Eur J Phys Rehabil Med](http://www.ncbi.nlm.nih.gov/pubmed?term=Zaina.%202009%20%20copd%20\%20# \ European journal of physical and rehabilitation medicine.) 2009;45:193-5.

9s. Seymour JM, Moore L, Jolley CJ, Ward K, Creasey J, Steier JS, Yung B, Man WD, Hart N, Polkey MI, Moxham J. Outpatient pulmonary rehabilitation following acute exacerbations of COPD. Thorax 2010;65:423-8.

10s. Lan CC, Yang MC, Lee CH, Huang YC, Huang CY, Huang KL, Wu YK. Pulmonary rehabilitation improves exercise capacity and quality of life in underweight patients with chronic obstructive pulmonary disease. Respirology 2011;16:276-83.

11s. Dodd JW, Hogg L, Nolan J, Jefford H, Grant A, Lord VM, Falzon C, Garrod R, Lee C, Polkey MI, Jones PW, Man WD, Hopkinson NS. The COPD assessment test (CAT): response to pulmonary rehabilitation. A multicentre, prospective study Thorax 2011;66:425-9.

12s. Clini EM, Crisafulli E, Costi S, Rossi G, Lorenzi C, Fabbri LM, Ambrosino N. Effects of early inpatient rehabilitation after acute exacerbation of COPD. Respir Med 2009;103:1526-31.

13s. Bratås O, Espnes GA, Rannestad T, Walstad R. [Pulmonary rehabilitation reduces depression and enhances health-related quality of life in COPD patients--especially in patients with mild or moderate disease.](http://www.ncbi.nlm.nih.gov/pubmed/21084547) Chron Respir Dis 2010;7:229-37.

14s. Steinsbekk A, Lomundal BK. [Three-year follow-up after a two-year comprehensive pulmonary rehabilitation program.](http://www.ncbi.nlm.nih.gov/pubmed/19176707) Chron Respir Dis 2009;6:5-11.

15s. Theander K, Jakobsson P, Jörgensen N, Unosson M. Effects of pulmonary rehabilitation on fatigue, functional status and health perceptions in patients with chronic obstructive pulmonary disease: a randomized controlled trial. Clin Rehabil 2009;23:125-36.

16s. Ninot G, Moullec G, Picot MC, Jaussent A, Hayot M, Desplan M, Brun JF, Mercier J, Prefaut C. [Cost-saving effect of supervised exercise associated to COPD self-management education program.](http://www.ncbi.nlm.nih.gov/pubmed/21036024) Respir Med 2011;105:377-85.

17s. Spencer LM, Alison JA, McKeough ZJ. Maintaining benefits following pulmonary rehabilitation: a randomised controlled trial. Eur Respir J 2010;35:571-7.

18s. Beaumont M, Reychler G, Le Ber-Moy C, Peran L. The effects of a pulmonary rehabilitation program in relation to the severity of COPD. Rev Mal Respir 2011;28:297-305.

19s. Hoogendoorn M, Feenstra TL, Hoogenveen RT, Rutten-van Mölken MP. Long-term effectiveness and cost-effectiveness of smoking cessation interventions in patients with COPD. Thorax 2010;65:711-8.

20s. Esteban C, Quintana JM, Aburto M, Moraza J, Egurrola M, Pérez-Izquierdo J, Aizpiri S, Aguirre U, Capelastegui A. Impact of changes in physical activity on health-related quality of life among patients with COPD. Eur Respir J 2010;36:292-300.
