# Supplementary material for: Simulation-Based Estimates of the Effectiveness and Cost-Effectiveness of Pulmonary Rehabilitation in Patients with Chronic Obstructive Pulmonary Disease in France
Source: PLoS One. 2016 Jun 21;11(6):e0156514. doi: 10.1371/journal.pone.0156514 (PMC4915708; doi:10.1371/journal.pone.0156514)
Supplement: S2 Table — (DOCX) [file pone.0156514.s003.docx]

S2 table : Parameter values use in the Marko simulation (for Initial age distributions (% of patients) of English COPD smoking patients according to severity, Probability of Death of a COPD patient according to age, smoking status, and severity, Transition probabilities from a severity stage to the next (see supportive information of reference 22)

| Parameter | Gold 1 | Gold 2 | Gold 3 | Gold 4 |
| --- | --- | --- | --- | --- |
| COPD severity distrubition (% of patients) | 35.08 | 48.17 | 13.6 | 2.79 |
| Annual smoking transition rates (% of patients)  Quit smoking  Resume smoking | 4.7  2.6 | 4.7  2.6 | 4.7  2.6 | 4.7  2.6 |
| Exacerbation rates (% of patients)  0 exacerbation  ≥ 1 exacerbation | 75  25 | 60.55  39.45 | 55.90  44.10 | 34.30  65.70 |
| Health utility (QALY)  0 exacerbation  ≥ 1 exacerbation | 0.897  0.985 | 0.755  0.736 | 0.749  0.726 | 0.549  0.535 |
| Annual cost of a COPD patients (euros) per year | 308 | 1016 | 5260 | 13258 |
